# Analysis of Cadmium Retention Mechanisms by a Smectite Clay in the Presence of Carbonates

**DOI:** 10.3390/toxics11020130

**Published:** 2023-01-29

**Authors:** Tiziana Missana, Ursula Alonso, Natalia Mayordomo, Miguel García-Gutiérrez

**Affiliations:** 1 CIEMAT, Physical Chemistry of Actinides and Fission Products Unit, 28040 Madrid, Spain; 2 HZDR, Institute of Resource Ecology, Helmholtz-Zentrum Dresden-Rossendorf, 01328 Dresden, Germany

**Keywords:** contaminants, cadmium, adsorption, surface complexation modelling, cation exchange, risk assessment, clays, geochemical barriers, otavite

## Abstract

Cadmium (Cd) is a toxic heavy metal with very low permissible exposure limits and is, thus, a very dangerous pollutant for the environment and public health and is considered by the World Health Organisation as one of the ten chemicals of major public concern. Adsorption onto solid phases and (co)precipitation processes are the most powerful mechanisms to retain pollutants and limit their migration; thus, the understanding of these processes is fundamental for assessing the risks of their presence in the environment. In this study, the immobilisation of Cd by smectite clay has been investigated by batch sorption tests, and the experimental data were interpreted with a thermodynamic model, including cation exchange and surface complexation processes. The model can describe the adsorption of Cd in smectite under a wide range of experimental conditions (pH, ionic strength, and Cd concentration). Under the conditions analysed in this study, the precipitation of otavite (CdCO_3_) is shown to have a limited contribution to Cd immobilisation.

## 1. Introduction

Cadmium is a hazardous contaminant that is toxic to humans and wildlife and represents a particularly important threat due to its toxicity at very low concentrations [1,2]. In the case of the presence of any contaminant in a potentially dangerous concentration, the application of the most adequate prevention or remediation strategies is necessary to minimise the associated risks [3,4].

Contaminant migration in the environment is mainly controlled by adsorption onto mineral surfaces, especially iron oxides, and clays, and by precipitation, these being the most efficient mechanisms to retain pollutants [5,6,7,8].

Different materials that are able to interact with the contaminants and retain them can be used in different types of waste disposals or employed for the containment of accidental discharges to prevent further Cd migration into the environment. Cd retention on several sorbents has been studied for years, considering either synthetic materials [9,10] or natural minerals and soils [11,12,13,14,15,16,17,18]. Clayey rocks are often used as barrier materials because they are long-lasting, environmentally safe, abundant, and cheap [19,20,21].

Thus, the in-depth analysis of adsorption processes is very important to design optimised sorbent materials, but the understanding of sorption mechanisms is also crucial for the development of mechanistic models, which are essential for the prediction of contaminant migration when the chemical conditions spatially and/or temporally change.

Most of the existing sorption studies perform quantitative evaluations of the absorbent efficiency under a short range of experimental conditions and often use Langmuir, Freundlich, or other types of empirical isotherms as a unique tool for data analysis. Instead, much less effort has been spent on the analysis of adsorption data by means of mechanistic sorption modelling, which, indeed, is the basis for the prediction of contaminant behaviour under variable conditions [22,23]. Recent studies have used multivariate competitive models [24] or molecular simulations [25] as descriptive tools, which are very useful as a support for the understanding of underlying surface processes.

The main objective of this work is to analyse Cd adsorption in smectite clay (FEBEX bentonite) [26] in the presence of carbonates, and to evaluate the most important retention mechanisms with the aid of adsorption modelling. The possible role of Cd precipitation on the overall retention behaviour is also discussed.

The role played by chloride on Cd adsorption must be analysed, too, as it is still controversial [27,28]. Crea et al. (2013) [29] reported that the soft character of Cd^2+^ implies a strong affinity towards soft ligands (e.g., Cl^−^), and this could affect Cd retention behaviour, as mentioned in the literature. In some cases, Cl^−^ was observed to induce a higher removal [30,31], while in others, it decreased the retention of absorbents [32,33,34]; thus, the relevance of chloride in Cd adsorption in our system has been also experimentally evaluated.

The main difference observed in Cd adsorption behaviour, with respect to that of other divalent metal ions in the same material, is underlined.

## 2. Materials and Methods

### 2.1. Smectite Clay

The clay used for this study is FEBEX bentonite. It was mined in Almeria (Spain), and it has been widely studied as a potential barrier material for high-level radioactive waste disposals [35,36]. This natural clay is mainly composed of smectite (93 ± 2%) with a minor quantity of accessory minerals, such as quartz (2 ± 1%), plagioclase (3 ± 1%), cristobalite (2 ± 1%), and calcite (<1%). The cation exchange capacity, CEC, of this clay is 100 ± 4 meq/100g, and its N_2_-BET surface area is 59 ± 2 m^2^·g^−1^ [23]. More details on its physical-chemical characteristics can be found elsewhere [26].

The clay was purified and converted into its Na-homoionic form using a standard methodology and will be referred to hereafter as Na-smectite. For the homoionisation process, the clay is suspended in 1 M NaClO_4_, the suspension is stirred overnight and decanted, and then the fresh electrolyte is added. This step is repeated three times to ensure that all soluble accessory minerals are eliminated, and the clay is converted into its Na-form. Then, the Na-smectite is placed in centrifuge tubes with ultrapure water. The fine fraction of the clay (<0.5 μm) is extracted by centrifuging the suspension (2500× *g*, 10 min) several times, taking the supernatant, and substituting the solution with fresh ultrapure water. Finally, the purified Na-smectite suspensions were brought to the desired ionic strength by dialysis with NaClO_4_ at different ionic strengths. The CEC and N_2_-BET surface area values of the purified Na-smectite fell into the range of the raw material. XRD measurements of the purified clay do not reveal the presence of minerals other than smectite.

The concentration of the Na-smectite in the suspensions was determined by gravimetry; the solid-to-liquid ratio used in these experiments was between 0.5 and 1 g·L^−1^ approximately.

### 2.2. Techniques Used for Cd Measurements

The analysis of Cd in the experiments was carried out by radiochemical techniques, which represent a fast and precise tool to determine the metal concentration at a trace level. The isotope used for this study was the ^109^Cd, in the form of CdCl_2_ in 1M HCl, which contained 37∙MBq·mL^−1^ and a CdCl_2_ carrier (500 µg Cd·mL^−1^) (Isotope Products). The half-life of ^109^Cd is 464 days; it decays by electron capture emitting photons of different energies: 0.088 MeV (4%), 0.025 MeV (18%), and 0.022 MeV (84%). Thus, its activity was measured by gamma counting with a Cobra-II auto-gamma Packard counter (NaI detector). For tests at a Cd concentration higher than 1 × 10^−6^, M aliquots of stable CdCl_2_ (Aldrich) were added with the radiotracer.

One specific test to was evaluate the possible effect of the electrolyte anion (Cl^−^ or ClO_4_^−^) on Cd retention, which was carried out using only stable Cd(ClO_4_)_2_ (Sigma-Aldrich, Darmstadt, Germany). In this test, Cd was measured by ICP-MS with a Thermo Fischer Scientific X SeriesII ICP-MS apparatus. Before the measurements, samples were acidified with HNO_3_ Suprapure (Sigma-Aldrich, Darmstadt, Germany).

Clay samples after the adsorption of Cd at high concentrations were analysed by the attenuated total reflection Fourier transform infrared spectroscopy (ATR-FTIR) with a Nicolet iS-50 apparatus in the range of 400–4000 cm^−1^ (middle infrared) to identify possible Cd precipitates.

### 2.3. Sorption Tests

Batch sorption experiments were carried out under atmospheric conditions and a temperature of 22 ± 3 °C. The effects of Cd concentration [Cd], ionic strength (I), and pH were analysed to provide a set of experimental data that were wide enough to develop and validate the sorption model. The kinetics of the sorption process was investigated first to determine the time required for the attainment of the sorption equilibrium.

Sorption edges (i.e., the sorption tests as a function of pH) were carried out by varying the pH of the suspensions from approximately pH 2 to 11 with NaOH or HCl 0.1, or 1 M. They were carried out at two ionic strengths (I = 0.1 and I = 0.01 M in NaClO_4_) and with [Cd] from 9 × 10^−8^ to 1 × 10^−5^ M.

To evaluate the potential effects of chloride in our experiments, additional adsorption edges were carried out in the absence of chloride (0.1 M NaClO_4_ and [Cd] = 1 × 10^−5^ M) using Cd(ClO_4_)_2_ instead of CdCl_2_ and acidulating with HClO_4_ instead of HCl. 

Adsorption isotherms were carried out, at two different pH values (5.1 ± 0.1 and 7.8 ± 0.1) and at a fixed background electrolyte concentration (0.1 M in NaClO_4_) using Cd concentrations from 2 × 10^−8^ M to 2 × 10^−3^ M, approximately.

The suspensions, at the desired pH and I, were introduced in centrifuge tubes, Cd was added, and the pH was readjusted after Cd addition. Tubes were stirred during the selected contact time, and then, the clay and the electrolyte were separated by centrifuging (25,000× *g*, 30 min) with a Thermo-Scientific MR23i centrifuge.

After the separation, three aliquots (2 mL) of the supernatant were extracted from each tube for the analysis of the final ^109^Cd activity by gamma measurements. The rest of the solution was used to check the final pH.

The degree of adsorption was expressed by the distribution ratio R_d_ [mL·g^−1^], defined as:

(1) $$R_{d}=\frac{C_{i}{}_{n}-C_{fin}}{C_{\begin{array}{c} fin \end{array}}}\cdot \frac{V}{m}$$

where *C_in_* and *C_fin_* are the initial and final concentrations of Cd in the liquid phase (Bq·mL^−1^), *m* is the mass of the clay (g), and *V* is the volume of the liquid (mL). Corrections must be made, when necessary when considering the adsorption onto the vials and centrifuge tubes. However, the adsorption of Cd onto these vessels was always lower than 1% and thus did not account for R_d_ estimations.

### 2.4. Sorption Modelling

To provide a deeper understanding of the effect of solution chemistry on contaminant retention, geochemical and thermodynamic modelling are very powerful tools. As a fundamental basis of sorption modelling, the speciation of the contaminant in the solution must be known, and this requires reliable values for the relevant stability constants of the species involved.

The most recent revision of the available thermodynamic data for Cd was performed by Powell et al. [37] for the International Union of Pure and Applied Chemistry (IUPAC). The reactions and constants used for the calculations are summarised in Table 1. The rest of the data used for calculations belongs to the EQ3/6 database [38] collected by the Lawrence Livermore National Lab (US).

Modelling calculations were carried out with the CHESS v 2.4 geochemical code [39]; therefore, all the reactions and constants given in the tables are expressed according to the notation of this code. 

For modelling Cd adsorption in the Na-smectite, we considered that cation adsorption in the clays could occur by two main different mechanisms: cation exchange and surface complexation on the amphoteric surface functional groups (SOH), which are present at the broken edges of the clay particles [40].

Clays have a structural and permanent negative charge, which is compensated by the adsorption of solute cations at their particle surface. The cation exchange is a process in which these cations can be exchanged by others present in the solution, thus maintaining the charge balance. This is a process independent of pH, but depending on I.

In the case of the Na-smectite, the existence of exchange sites (X=Na) with a density equivalent to the CEC of the clay can be assumed. The Na exchange with Cd is defined by the following equation:

2(X=Na) + Cd^2+^ ⇔ X_2=_Cd + 2 Na^+^ (2)

This equilibrium is expressed by a selectivity coefficient, KNaCdSEL, that can be calculated following the Gaines–Thomas notation [41] using this expression [42]:

(3) KNaCdSEL=RdexcCEC·γNa2γCd(Na)2

where γ_Na_ and γ_Cd_ are the solution activity coefficients of cations Na and Cd and R_d_(exc) is the distribution coefficient due to ion exchange contribution. CEC is the cation exchange capacity of the clay, and (Na) is the concentration of the main electrolyte cation.

The corrections for ionic strengths have been calculated with Davies’ approximation.

According to theory, for a 2:1 cation exchange process, the dependence of Cd adsorption expressed as Log(R_d_(exc)) should be linear with the logarithm of the cation concentration in the electrolyte, Log(Na); thus, the slope of this curve should be −2 [42].

Surface complexation on SOH sites usually depends significantly on the pH because SOH sites can be protonated or deprotonated according to these reactions:

SOH_2_^+^ ⇔ SOH + H^+^ (4)

SOH ⇔ SO^−^ + H^+^ (5)

Two different types of adsorption sites, weak (S_w_OH) and strong (S_s_OH), have been reported for cation adsorption studies in the FEBEX clay [23], and their protonation and deprotonation constants are summarised in Table 2. The densities of the (S_w_OH) and strong (S_s_OH) sites for this clay are 1.02 and 3.4 × 10^−2^ μeq·m^2^ [23].

Metal ions can interact with both SOH sites, forming different types of complexes, depending on the aqueous speciation and the hypothesis of the model. The general expression for inner sphere monodentate complexation for a generic cation M of valence *z* is:

SOH + M^z+^ ⇔ SOM^z−1^ + H^+^ (6)

For Cd^2+^ complexation, it is:

(6b) $$SOH+Cd^{2+}\Leftrightarrow SOCd^{+}+H^{+}$$

The adsorption model used in this study to represent Cd retention accounts for cation exchange and the existence of two different complexation sites at the smectite surface. The surface complexation model is not electrostatic [23].

## 3. Results

### 3.1. Cd Speciation

Calculations of Cd speciation were performed as a function of the pH of the solution and two different Cd concentrations. To be useful for the interpretation of the experimental adsorption data, these calculations must contain information on the effects produced by pH and Cd concentration, but also by the presence of aqueous ions and relevant ligands in the solution.

Adsorption tests were carried out under atmospheric conditions; therefore, the formation of carbonate complexes in the solution was expected. The content of carbonates was measured in several samples used for the adsorption tests, and the mean value at pH = 7 was expressed as [HCO_3_^−^], is (1.0 ± 0.1) × 10^−3^ M. This value was used for the calculations to account for the possible formation of Cd-carbonate species.

In our system, chloride can be introduced with HCl, which is usually used to change the pH of the suspensions by the addition of Cd in the form of CdCl_2_. The maximum possible concentration of the anion in our system was estimated to be around 1 × 10^−3^ M, thus calculations were performed considering the maximum concentration of chloride.

**Figure 1:**
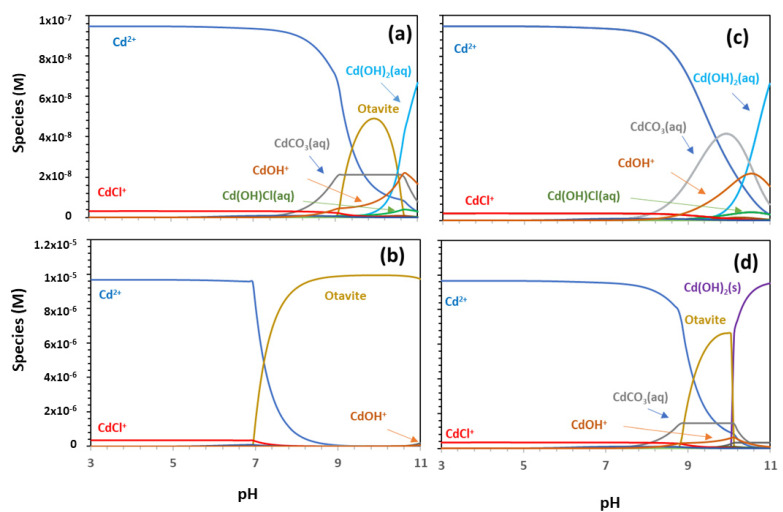
Cd speciation as a function of pH. Calculations were made considering NaClO_4_ 0.1 M and in the presence of [Cl^−^] = 1 × 10^−3^ M and 1 × 10^−3^ M carbonates (as HCO_3_^−^). For (**a**,**c**) the Cd concentration is 1 × 10^−7^ M and for (**b**,**d**) the Cd concentration is 1 × 10^−5^ M. The thermodynamic constants used are from Powell et al. [37]. In Figures (**c**,**d**), the value for otavite was considered to be −0.1 instead of 1.7336 [37].

The results of the calculations for Cd speciation, as a function of pH and in the presence of chloride and carbonates are shown in Figure 1. Figure 1a,b correspond to the calculations obtained regarding the two different concentrations of Cd (1 × 10^−7^ M and 1 × 10^−5^ M) and using the constants of Table 1.

Figure 1c,d represents the calculations at the same Cd concentrations and the same thermodynamic constant with the unique difference for the formation constant of the solid otavite (CdCO_3_). This constant was adjusted according to our experimental adsorption data; this issue is discussed later, considering the values and uncertainties reported for this constant [37,43,44].

Figure 1a shows that, at the lower Cd concentration, the main species in the solution for pH < 9 was Cd^2+^, the CdCO_3_(aq) species started to be present after pH 8, and the species Cd(OH)_2_(aq) and Cd(OH)^+^ were present after pH 9. According to the data of Powell et al. [37], the calculation predicts the precipitation of CdCO_3_ (s) (otavite) between pH 9 and 10.5, even at this low Cd concentration.

The main Cd-Cl species observed in the speciation diagram are CdCl^+^ at pH<9 and Cd(OH)Cl(aq) at pH > 9. The effect of chloride on Cd speciation is not negligible, but the total amount of Cd-Cl species did not exceed 5% of the total Cd.

At the Cd concentration of 1 × 10^−5^ M, up to pH 7.3, the main aqueous species is Cd^2+^. At pH > 8, the code predicts the complete precipitation of Cd in the form of otavite, which should then play, under alkaline conditions, an important role in Cd retention (even at low concentrations). Additionally, in this case, CdCl^+^ is the Cd-Cl species observed under an acidic pH, with a contribution of less than 5%. Other minor species obtained in the calculations are not specifically identified in the graphs.

Considering the results of the calculations (Figure 1a,b), the main aqueous species to be considered in adsorption modelling is Cd^2+^, which dominates the speciation under acidic and slightly alkaline conditions. Other charged species, which might be coordinated to the surface, and which appear in the speciation diagram, although in a much smaller quantity, are CdCl^+^ and Cd(OH)^+^.

Finally, the possible precipitation of otavite must also be analysed as a possible retention mechanism, as it is predicted at a very low Cd concentration.

### 3.2. Sorption Tests

**Figure 2:**
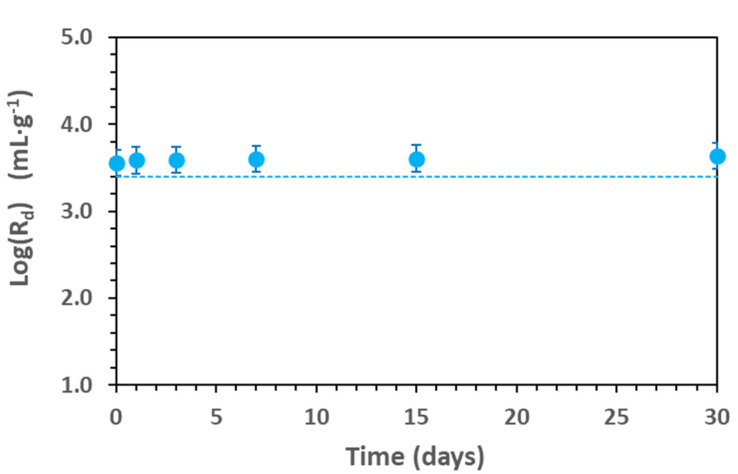
Cd adsorption kinetics in Na-smectite (0.6 g·L^−1^). [Cd] = 9.2 × 10^−8^ M; pH = 7 in 0.1 M NaClO_4_. The line represents the model calculation with the parameters of Table S1.

Figure 2 shows the adsorption kinetics of Cd in Na-smectite, ([Cd] = 9.2 × 10^−8^ M; 0.1 M NaClO_4_ and pH = 7 ± 0.1) measured for 30 days.

The results are expressed as the logarithm of the distribution coefficient (Equation (1)), Log(R_d_), and show that the adsorption equilibrium was reached within hours and that R_d_ remained stable over time. The time selected for all the other adsorption tests was 7 days.

**Figure 3:**
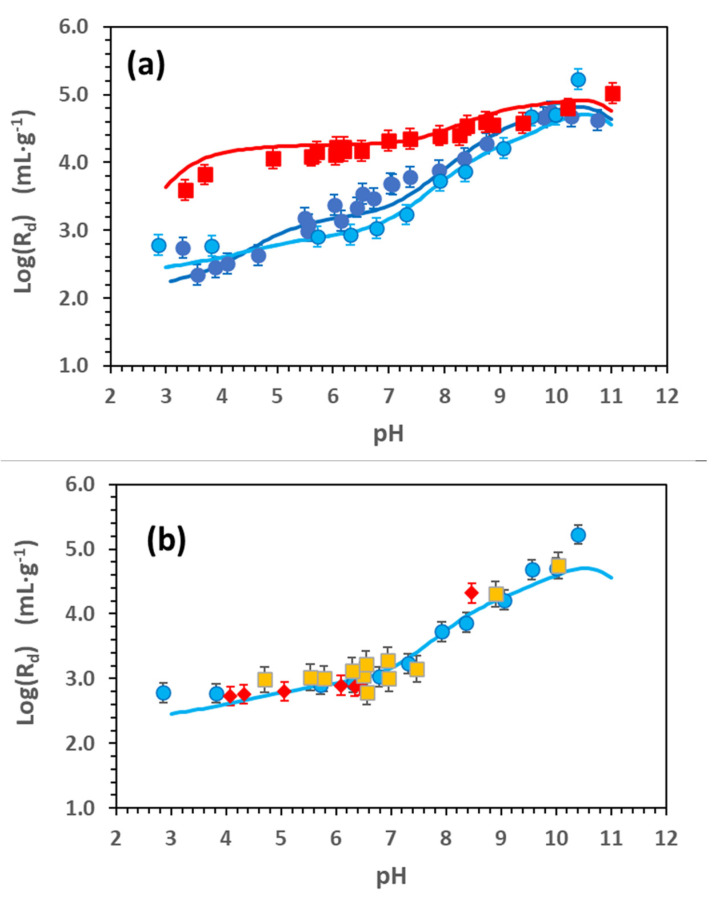
Cd adsorption edges in Na-smectite [0.57–0.86 g·L^−1^]. (**a**) (⬤) Comparison of sorption edges at two different Cd concentrations. [Cd] = 9.18 × 10^−8^ M and 0.1 M NaClO_4_; (⬤) [Cd] = 1 × 10^−5^ M and 0.1M NaClO_4_; (◼) [Cd] = 9.18 × 10^−8^ M and 0.01M NaClO_4_. (**b**) Comparison of sorption edges with different contents of chloride. (⬤) Reference standard test (Cd] = 1 × 10^−5^ M with HCl and CdCl_2_); (◆) [Cd] = 1 × 10^−5^ M with Cd(ClO_4_)_2_ and traces of ^109^CdCl_2_ (1 × 10^−8^ M); (◼) [Cd] = 1 × 10^−5^ M without Cl (only HClO_4_ and Cd(ClO_4_)_2_. The line represents the model calculation with the parameters of Table S1.

Figure 3 includes the results of Cd adsorption edges (Log(R_d_) vs. pH). Figure 3a shows the tests carried out at two different ionic strengths in NaClO_4_ (0.1 and 0.01) at [Cd] = 9.18 × 10^−8^ M; a third experiment was carried out in 0.1 M NaClO_4_ and a higher Cd concentration ([Cd] = 1 × 10^−5^ M) to better elucidate the contribution of strong and weak surface sites. Figure 3b compares the adsorption edges carried out in 0.1 M NaClO_4_ ([Cd] = 1 × 10^−5^ M) and carried out with different chloride concentrations.

The clear dependence of adsorption on pH and I can be observed in Figure 3a. The Log(R_d_) for Cd presents a dependence on ionic strength; the higher the ionic strength, the lesser the adsorption, especially under acidic conditions.

On the other hand, data at the highest ionic strength (0.1 M) present a significant dependence on pH, which is instead minimal at 0.01 M. At 0.01 M, a slight decrease in adsorption could be seen at pH < 4. This effect could be related to some dissolution of the clay but also to the exchange reaction between sodium and the proton, which, with the selectivity coefficient in the FEBEX clay, KNaHSEL, was between 0.7 and 1. This exchange process is accounted for in the calculations.

Some differences could also be observed between the data obtained at I = 0.1 M for the two different Cd initial concentrations (Figure 3a). At the higher Cd concentration, a change in the slope of Log(R_d_) can be observed at pH > 10, which could be related to some Cd precipitation. No signals of precipitation are observed in the adsorption edges obtained at the lower Cd concentration.

Another difference is that the contribution of cationic exchange is much more evident in the sorption edge obtained with a high Cd concentration because the region in which the adsorption is almost independent of pH extends from pH 3 to 7, whereas it is scarcely visible in the other case.

All these observations indicate that Cd retention in Na-smectite is controlled by more than one mechanism and that one or another can prevail depending on the initial conditions of the experiment.

Figure 3b shows that the adsorption edges were carried out to analyse the effect of chloride on Cd retention ([Cd] = 1 × 10^−5^ M) by the Na smectite. One test was carried out in the absence of chloride by using stable Cd(ClO_4_)_2_ as a tracer and modifying the pH with HClO_4_. The second test was performed under the same conditions but by adding a small quantity of radioactive ^109^CdCl_2_, (1 × 10^−8^ M). These data are compared with the adsorption edges performed with the standard experimental conditions.

All the adsorption edges obtained with a different Cl^−^ content is very similar, demonstrating that the effect of chloride in Cd retention is negligible under the conditions of the present experiments. Even if there was a quantity of Cl^−^ in the system that might be as high as 1 × 10^−3^ M, a correct definition of the chemical system (Figure 1a,b) must be accounted for where the contribution of Cl-Cd species for adsorption modelling can be disregarded.

**Figure 4:**
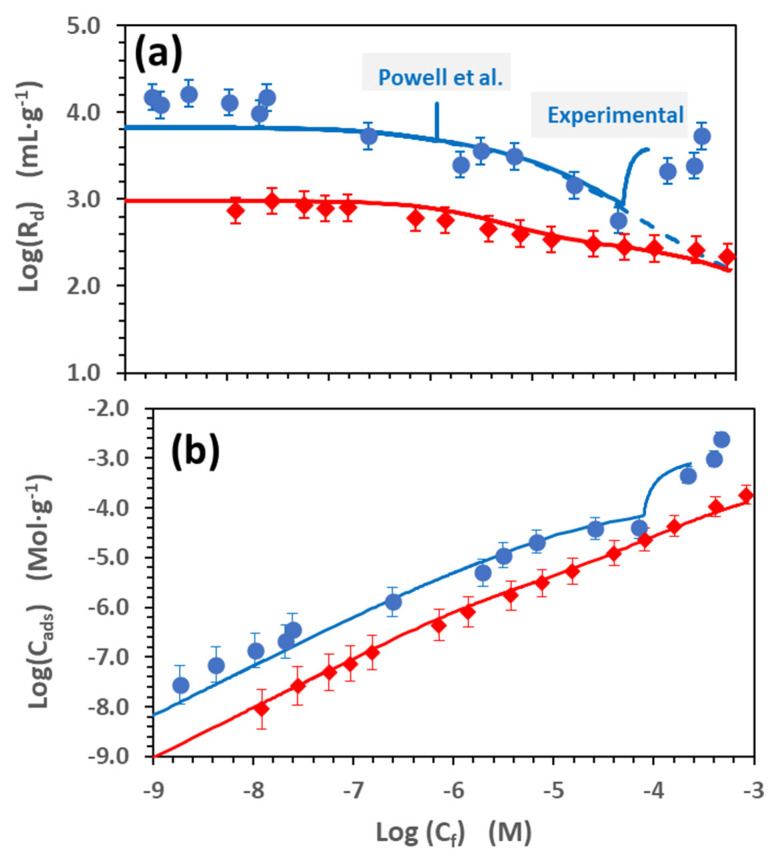
Cd adsorption isotherms in Na-smectite in 0.1 M NaClO_4_. (**a**) data expressed as Log(R_d_) vs. the logarithm of Cd final concentration at the equilibrium, Log(C_f_), and (**b**) data expressed as the logarithm of the Cd adsorbed per unit mass (Log (C_ads_) vs. Log(C_f_). (⬤) pH = 7.8 and (◆) pH 5.1. The lines represent the model calculation with the parameters of Table S1. The concentrations where precipitation is predicted by the Powell et al. database [37] or were experimentally observed are highlighted in Figure 4a.

Figure 4 shows the Cd sorption isotherms obtained at two different pH values (7.8 and 5.1). Figure 4a shows the data expressed as the logarithm of the distribution ratio, Log(R_d_) vs. the logarithm of the final Cd concentration at the equilibrium, Log(C_f_), in [mol·L^−1^] and Figure 4b as the logarithm of the Cd adsorbed for the unit mass of the absorbent Log(C_ads_) in [mol·g^−1^]

The isotherms show a “linear zone” at low Cd concentrations, where R_d_ is approximately constant; then, as the Cd concentration increases, R_d_ values start decreasing due to the saturation of the adsorption sites. Nevertheless, in the adsorption isotherm at pH 7.8, a sudden increase in the R_d_ is observed when the Cd aqueous concentration exceeds 8 × 10^−5^ M, approximately. This deviation from the theoretical behaviour, which corresponds to a slope higher than one in the Log(C_ads_) vs. Log(C_f_) graph (Figure 4b), is usually related to precipitation processes. At pH 5.1, no signs of precipitation could be appreciated at any Cd concentration.

## 4. Discussion

The predominant adsorption mechanism in the FEBEX-clay for alkaline cations (Cs^+^, Ca^2+^, Sr^2+^) [23,40,45,46,47,47] is cation exchange (Equation (2)), and for these elements, the adsorption is highly dependent on the ionic strength of the solution, but almost independent of the pH up to pH 9 or even higher.

The adsorption behaviour of ions such as UO_2_^2+^ [48] or Eu^3+^ [23] is different; the region where the R_d_ is independent of the pH and dependent on ionic strength, which is attributable to cation exchange, is limited to the (very) acidic range of the pH. As the pH increases, adsorption starts depending on pH (and much less on I), and this behaviour can be assigned to the inner sphere surface complexation. Cation exchange and surface complexation can coexist, and their relative relevance depends on the type of contaminant and on the chemical conditions.

The dependence on pH and electrolyte concentration for Cd adsorption in Na-smectite, as shown in Figure 3a, indicates that Cd sorption must also be controlled by more than one adsorption process.

Therefore, preliminary modelling calculations were carried out considering the contribution of ionic exchange (Equation (3)) and surface complexation (Equation (6b)), adsorbing only Cd^2+^: the principal ion in the solution. The possible adsorption of CdCl^+^ was discarded at the beginning due to the results shown in Figure 3b. With this initial approach successfully used in other cases [23,45], the simulation of the experimental Cd adsorption data were quite poor because the dependence of adsorption with the pH could not be reproduced using only Equation (6b) (results not shown).

To explain the surface complexation of Cd in goethite, Gunneriusson [49], in additional to the reaction expressed in (Equation (6b)) proposed the following reactions:

(7) $$SOH+Cd^{2+}\Leftrightarrow SOHCd^{2+}$$

(8) $$SOH+CdOH^{+}\Leftrightarrow SOCdOH+2H^{+}$$

Equation (7) implies the complexation of Cd on the neutral SOH site, and Equation (8) describes the surface complexation of the first Cd hydrolysed species on the (deprotonated) negatively charged site.

These reactions have been tested, accounting for both the strong and weak surface sites of the FEBEX clay. All the tests presented in Figure 3 and Figure 4 were separately simulated, and all the parameters used in each case to fit the experimental data are reported in Table S1 of the Supplementary Material, indicating the mean value for each constant and the standard deviation.

Table 3 collects the reactions defining the different surface complexes, according to the Chess code, and the corresponding mean values of the constants (LogK).

The fit obtained with the model parameters is superimposed as a continuous line to the experimental points in Figure 1, Figure 2 and Figure 3. An example of the contribution of each surface complex to the total Cd retention is given in the Supplementary Material (Figure S1).

This model reproduces very well the Cd sorption behaviour under the range of pH, ionic strength, and Cd concentrations analysed.

Furthermore, all the tests could be consistently fit, basically with the same parameters and with a maximum standard deviation of 0.16 (Table S1), which is compatible with the experimental error.

It could also be concluded that the second reaction proposed for the surface complexation by Gunneriusson [49], making a surface complex with the first hydrolysed Cd species (Equation (8)), allowed the fitting the experimental data at pH > 10, which was not affected by precipitation, but did not really improve the overall simulation of the curves. For the sake of simplicity, this complex could be overlooked.

The logarithm of the selectivity coefficient, KNaCdSEL , for Na-Cd exchange (Equation (3)) derived from the present study is 0.8 ± 0.16, which is a value totally comparable with the selectivity coefficients of other divalent cations in the same clay [45]. However, while the exchange process for Cd seems to be equivalent to other divalent cations, surface complexation is not.

The need to introduce another complexation reaction (Equation (7)), in addition to the usual inner sphere complexation reaction (Equation (6b)), is an indication that the Cd^2+^ complexation, under an acid-neutral pH, is more complex than that reported for other divalent cations such as Ca^2+^ or Sr^2+^, which also dominate the speciation in the system, and are mostly retained by the ionic exchange in a wider range of pH.

**Figure 5:**
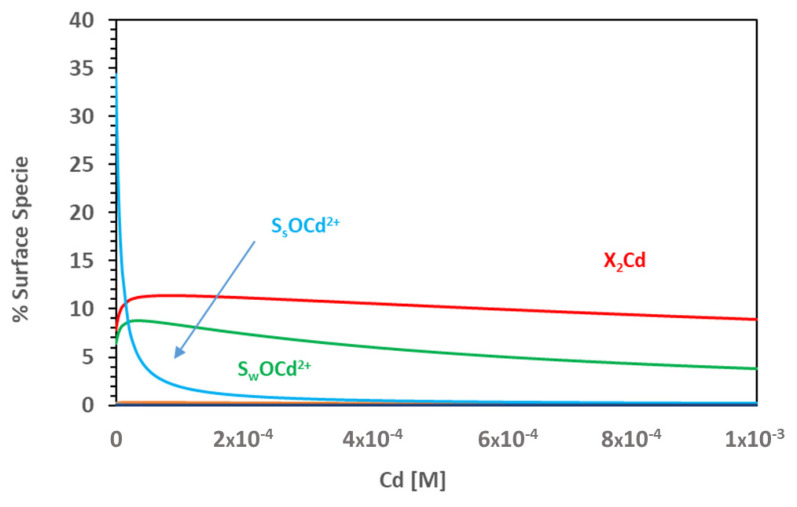
Percentage of surface species formed as a function of Cd concentration. 0.1 M NaClO_4_ and pH = 5.1.

For example, through the simulation of the sorption isotherms, is it possible to analyse the importance of the Cd concentration on the relevance of the different contributions to Cd retention by Na-smectite. Figure 5 shows the Cd surface speciation at pH 5.1, using the data of the simulation of the isotherm (Figure 4).

At a Cd concentration lower than 1 × 10^−5^ M, the predominant surface complex is the strong S_s_OCd^2+^; only when [Cd] is higher than 1 × 10^−5^ M does the exchange complex, X_2_Cd, start predominating. This means that, at pH values below seven, the contribution of ionic exchange to Cd sorption by Na-smectite is dependent on the Cd concentration. This explains the different dependence on the pH observed and the shape of the adsorption edge at 0.1 M, which was performed with two different Cd concentrations (Figure 3a).

The results from the adsorption isotherm at pH 7.8 (Figure 4) raise another relevant issue. The shape of the isotherm indicates that precipitation occurs when the Cd concentration in the solution is higher than 8 × 10^−5^ M. If the simulations of the adsorption isotherms are performed with the thermodynamic data of Table 1, the precipitation of the Cd carbonate otavite (CdCO_3_) is predicted at a Cd concentration that is two orders of magnitude lower (around 1 × 10^−6^ M). ATR-FTIR analysis was carried out on the initial Na-smectite samples and upon Cd (3 × 10^−3^ M) adsorption at pH = 8 (Figure S2 in the Supplementary Material). In the sample with Cd, some changes were observed, which are consistent with the precipitation of the otavite. In fact, the main peaks appearing in the clay at approximately 1410 cm^−1^ and 860 cm^−1^, after the contact with Cd, coincided with the main peaks of otavite.

Otavite solubility has been experimentally analysed in many aqueous systems [43,44], and solubility products vary from values <1 proposed by Stipp et al. [44] to values of up to 1.73, proposed by Powell et al. [37]. These studies considered very different electrolytes from highly diluted to brines or marine waters. Furthermore, this solubility product is reported to be also affected by the actual partial pressure of CO_2_(g) and by the presence of other cations [43], leading to an averaged thermodynamic constant with relatively high uncertainty.

According to the data presented in Figure 4, the solubility of the otavite is almost two orders of magnitude lower than initially predicted. Thus, the solubility product was re-valuated to reproduce accordingly the experimental data, and the value obtained was −0.1. Considering this recalculated value and all the equilibrium reactions included in Table 1, the Cd speciation was recalculated (Figure 1c,d).

The decrease in this constant corresponded to an increase in Cd solubility, leading to relevant changes in Cd speciation in the alkaline region. At low Cd concentrations, no precipitation is predicted, as the aqueous CdCO_3_(aq) is formed, which agrees better with our experimental sorption results. At high Cd concentrations, the precipitation of otavite shifted from pH 7 to 9.5, and the range of its predominance was much narrower. Additionally, the hydroxide Cd(OH)_2_ (s) could also contribute to Cd precipitation at pH > 10.

Thus, the role of otavite precipitation seems to not be relevant to Cd retention by Na-smectite under the conditions analysed in the present study, and the aqueous Cd, which was eventually mobile in the environment, was substantially higher than the initially predicted, considering otavite precipitation.

## 5. Conclusions

Cadmium retention by smectite can be described considering two sites (weak and strong) of a non-electrostatic complexation model plus cation exchange. To provide the best fit of sorption data as a function of the pH, two species were considered: SOCd^+^ and SOHCd^2+^, corresponding to Cd coordination with the deprotonated and neutral adsorption SOH site of the clay, respectively. The latter complex is mainly present under acidic-neutral conditions and competes with cation exchange, especially at a low Cd concentration, and this may hamper the determination of the Na-Cd selectivity coefficient for cation exchange. The logarithm of the selectivity coefficient, KNaCdSEL , for the Na-Cd exchange estimated in this study was 0.8 ± 0.16.

For what concerns the presence of carbonates, according to speciation calculations, is the precipitation of otavite (CdCO_3_) at a Cd concentration higher than 1 × 10^−7^ M, which would be the main phenomena controlling Cd solubility and retention in the system under alkaline conditions. However, experimental sorption isotherms revealed signals of Cd precipitation only at a Cd concentration two orders of magnitude higher. Therefore, the overall Cd mobility would be higher than initially predicted.

## Figures and Tables

**Table 1:** Thermodynamic constants for Cd, taken from [37]. Data with (*) had a large uncertainty or values were not specifically suggested in [37]; thus, the available data in the EQ3/6 database were used.

Aqueous Species	Definition	LogK
CdOH [+]	1 Cd [2+], 1 H_2_O, -1 H[+]	−9.91 ± 0.1
Cd(OH)_2_(aq)	1 Cd [2+], 2 H_2_O, -2 H[+]	−20.19 ± 0.13
Cd(OH)_3_[−]	1 Cd [2+], 3 H_2_O, -3 H[+]	−33.5 ± 0.5
Cd(OH)_4_[2−]	1 Cd [2+], 4 H_2_O, -4 H[+]	−47.28 ± 0.15
Cd_2_OH [3+]	2 Cd [2+], 1 H_2_O, -1 H[+]	−8.73 ± 0.01
Cd_4_(OH)_4_[4+]	4 Cd [2+], 4 H_2_O, -4 H[+]	−31.8
CdHCO_3_[+]	1 Cd [2+], 1 HCO_3_[−]	0.84–2.4/1.5 (*)
CdCO_3_(aq)	1 Cd [2+], 1 HCO_3_[−], -1 H[+]	−5.9288
Cd(CO_3_)_2_[2−]	1 Cd [2+], 2 HCO_3_[−], -2H[+]	−14.4576
CdCl[+]	1 Cd [2+], 1 Cl[−]	1.98 ± 0.06
CdCl_2_(aq)	1 Cd [2+], 2 Cl[−]	2.64 ± 0.09
CdCl_3_[−]	1 Cd [2+], 3 Cl[−]	2.3 ± 0.21
Cd(OH)Cl(aq)	1 Cd [2+], 1 Cl[−], 1 H_2_O, -1 H[+]	7.4328 (*)
Solid Species	Composition	LogK
Cd(OH)_2_	1 Cd [2+], -2 H[+], 2 H_2_O	−13.72 ± 0.12
CdCO_3_(s), otavite (**)	1 Cd [2+], 1 HCO_3_[−], -1 H[+]	1.7336
Cd(OH)Cl	1 Cd [2+], 1 Cl[−], -1 H[+], 1H_2_O	−3.543
CdCl2	1 Cd [2+], 2 Cl[−]	0.674 (*)
CdCl_2_:H_2_O	1 Cd [2+], 2 Cl[−], 1 H_2_O	1.6747 (*)

** The value given for the otavite in [27] is discussed in the text.

**Table 2:** Properties of the surface functional groups of the clay used in this study. The reactions and constants are expressed according to the CHESS code used for calculating them [23].

SPECIES	DEFINITION	LogK
S_w_OH_2_[+]	1 S_w_OH, 1 H[+]	5.3
S_s_OH_2_[+]	1 S_s_OH, 1 H[+]	4.8
S_w_O [−]	1 S_w_OH, -1 H[+]	−8.4
S_s_O [−]	1 S_s_OH, -1 H[+]	−9.9

**Table 3:** Summary of the reactions and mean LogK used for simulating the set of data following the nomenclature of the CHESS code. The constants obtained in each test are detailed in Table S1.

SPECIES	DEFINITION	Mean LogK
X_2_Cd	2 X-Na, 1 Cd [2+], -2 Na[+]	3.5
S_w_OCd[+]	1 S_w_OH, -1 H[+], 1 Cd [2+]	−2.51
S_s_OCd[+]	1 S_s_OH, -1 H[+], 1 Cd [2+]	−1.4
S_w_OHCd [2+]	1 S_w_OH, 1 Cd [2+]	4.14
S_s_OHCd [2+]	1 S_s_OH, 1 Cd [2+]	6.1
S_s_OCdOH	1 S_w_OH, -2 H[+], 1 Cd [2+], 1 H_2_O	−11.66
S_w_OCdOH	1 S_s_OH, -2 H[+], 1 Cd [2+], 1 H_2_O	−11.86

## Data Availability

Data can be provided by the authors on request.

## Supplementary Materials

The following supporting information can be downloaded at: https://www.mdpi.com/article/10.3390/toxics11020130/s1, Table S1: Parameters used to simulate each experimental curve, presented in Figure 3 and Figure 4. Figure S1: Contribution to the final adsorption of the different surface species for the data in Figure 2b. Figure S2: Comparison of the ATR-FTIR spectra of Na-smectite before and after the adsorption of Cd [2 × 10^−3^ M] at pH = 8. The ATR-FTIR spectrum of otavite is also shown.
