# Supplementary material for: Analysis of Cadmium Retention Mechanisms by a Smectite Clay in the Presence of Carbonates
Source: Toxics. 2023 Jan 29;11(2):130. doi: 10.3390/toxics11020130 (PMC9959410; doi:10.3390/toxics11020130)
Supplement: Supplementary file 1 [file toxics-11-00130-s001.zip › toxics-2154459-supplementary.pdf]

toxics

MDPI

Supplementary Materials: Analysis of Cadmium Retention Mechanisms by a Smectite Clay in the Presence of Carbonates

Tiziana Missana, Ursula Alonso, Natalia Mayordomo and Miguel García-Gutiérrez

Table S1. Parameters used to simulate each experimental curve, presented in Figure 3 and Figure 4.

TEST	X2Cd	SOwCd[+]	SOsCd[+]	SOwCd[2+]	SOsCd[2+]	SOsCdOH	SOwCdOH
EDGE, 0.1 M Low Cd	3.50	-2.50	-1.40	4.30	6.10	-11.70	-11.90
EDGE, 0.01 M Low Cd	3.70	-2.55	-1.40	4.10	6.10	-11.50	-11.70
EDGE, 0.1 M High Cd	3.30	-2.50	-1.40	4.10	6.10	-11.70	-11.90
IT, 0.1 M pH=7.8	3.60	-2.50	-1.40	4.10	6.10	-11.70	-11.90
IT, 0.1 M pH=5.2	3.40	-2.50	-1.40	4.10	6.10	-11.70	-11.90
**MEAN**	**3.50**	**-2.51**	**-1.40**	**4.14**	**6.10**	**-11.66**	**-11.86**
**Desvest**	**0.16**	**0.02**	**0.00**	**0.09**	**0.00**	**0.09**	**0.09**

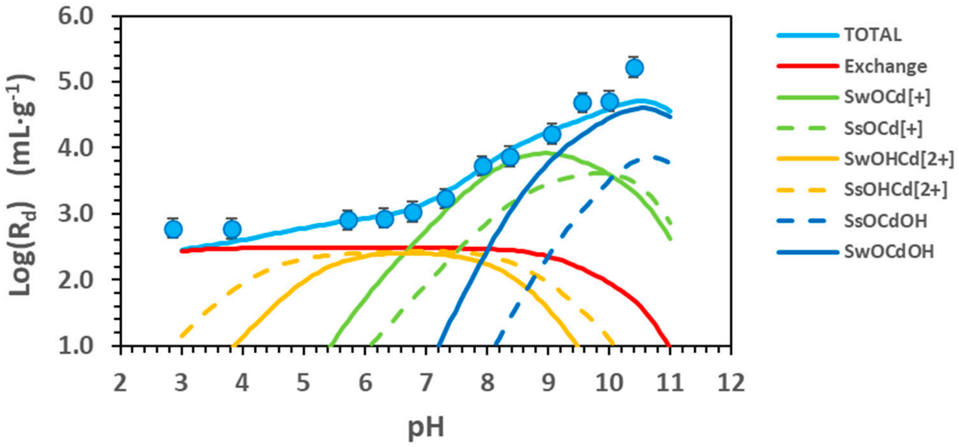

Figure S1. Contribution to the final adsorption of the different surface species for the data in Figure 2b.

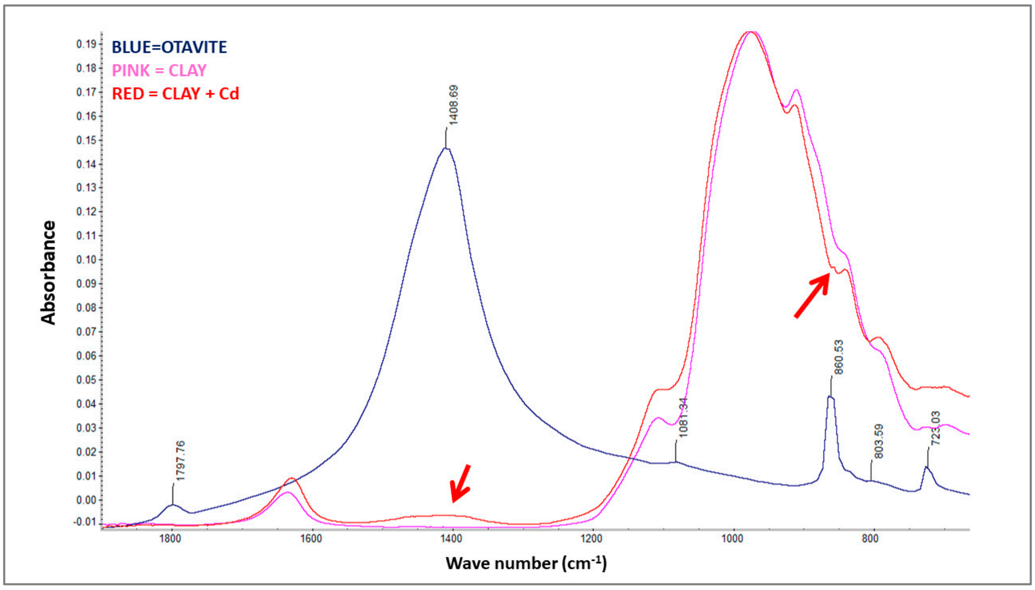

Figure S2. Comparison of the ATR-FTIR spectra of Na-smectite before and after the adsorption of Cd [3·10^{-3} M] at pH=8 in $NaClO_4$ 0.01 M. The reference spectra of otavite is also included for comparison.
